# Turning On Lights to Stop Neurodegeneration: The Potential of Near Infrared Light Therapy in Alzheimer's and Parkinson's Disease

**DOI:** 10.3389/fnins.2015.00500

**Published:** 2016-01-11

**Authors:** Daniel M. Johnstone, Cécile Moro, Jonathan Stone, Alim-Louis Benabid, John Mitrofanis

**Affiliations:** ^1^Department of Physiology, University of SydneySydney, NSW, Australia; ^2^University Grenoble Alpes, CEA, LETI, CLINATEC, MINATEC CampusGrenoble, France; ^3^Department of Anatomy, University of SydneySydney, NSW, Australia

**Keywords:** disease-modifying, neuroprotection, photobiomodulation, amyloid plaques, tau protein

## Abstract

Alzheimer's and Parkinson's disease are the two most common neurodegenerative disorders. They develop after a progressive death of many neurons in the brain. Although therapies are available to treat the signs and symptoms of both diseases, the progression of neuronal death remains relentless, and it has proved difficult to slow or stop. Hence, there is a need to develop neuroprotective or disease-modifying treatments that stabilize this degeneration. Red to infrared light therapy (λ = 600–1070 nm), and in particular light in the near infrared (NIr) range, is emerging as a safe and effective therapy that is capable of arresting neuronal death. Previous studies have used NIr to treat tissue stressed by hypoxia, toxic insult, genetic mutation and mitochondrial dysfunction with much success. Here we propose NIr therapy as a neuroprotective or disease-modifying treatment for Alzheimer's and Parkinson's patients.

## Introduction

Several recent studies in animal models of Alzheimer's and Parkinson's disease have reported that low-level near infrared light (NIr) therapy not only mitigates the behavioral deficits associated with these conditions but also has neuroprotective effects, slowing the underlying death of neurons. Current clinical therapies for both diseases do not achieve a comparable slowing of degeneration and neuroprotection, though they do relieve motor signs in Parkinson's disease and, to a lesser extent, the cognitive, and memory deficits in Alzheimer's disease. In this review, we consider the evidence for neuroprotection by NIr in animal models of these diseases, the putative mechanisms by which NIr may work to protect cells against insult, the safety of NIr therapy and finally, the potential effective use of NIr therapy in patients. First, we provide an overview of Alzheimer's and Parkinson's disease and current treatment options for these conditions.

## Overview and current treatment options for Alzheimer's and Parkinson's disease

Neurodegeneration refers to a progressive death of neurons, by either genetic environmental or currently unknown factors. It includes a range of disorders, with the two most common being Alzheimer's and Parkinson's disease. Over time, as more and more neurons die, the signs and symptoms associated with each disorder worsen, making many routine day-day activities increasingly more difficult for patients (Tierney et al., 2013; Schapira et al., 2014; Brettschneider et al., 2015; Coppedè and Migliore, 2015; Goedert, 2015; Herrup, 2015; Nelson and Tabet, 2015). In the sections that follow, the different patterns of neurodegeneration, clinical syndromes and current treatments for each disease will be considered separately.

### Alzheimer's disease

**Figure 1:**
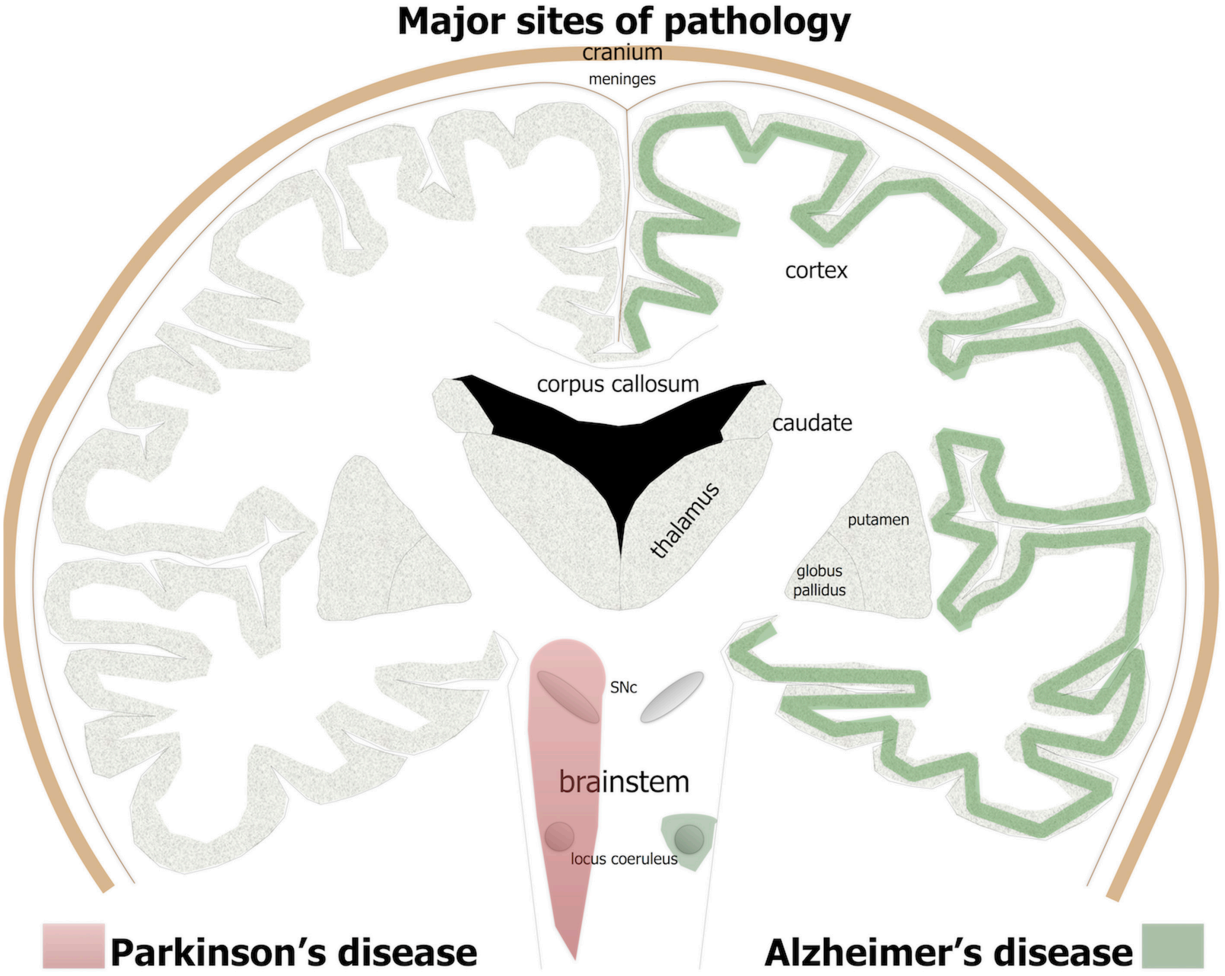
**The major brain sites of pathology in Alzheimer's and Parkinson's patients**. For Alzheimer's disease, green shade indicates major regions of cell loss and β-amyloid plaques and tau pathology, while in Parkinson's disease, red shade indicates sites of major cell loss and α-synuclein pathology.

Alzheimer's disease is the name given to an age-related, insidious-onset, progressive dementia. Individuals suffer progressive memory and cognitive decline and an overall loss of executive function (Herrup, 2015; Nelson and Tabet, 2015). There is an insidious death of neurons across large areas of the brain (Figure 1); all cortical regions, in particular entorhinal cortex and hippocampus, together with some subcortical regions, including the basal nucleus of Meynert, dorsal raphe, and locus coeruleus, suffer extensive neuronal death (Goedert, 2015; Herrup, 2015). The disease gained its name after the German neurologist Alois Alzheimer (1907, 1911) described three features of the end-stage brain. Two of the three features are proteinopathies (of β-amyloid and hyperphosphorylated tau); the third is now called gliosis or inflammation. Many other abnormalities have since been described in the dementing brain, from small vessel hemorrhage to oxidative stress (see below).

The Alzheimer's brain is characterized by a distinct pathology featuring numerous extracellular β-amyloid plaques and intracellular neurofibrillary tangles. The β-amyloid peptide, forming the bulk of the plaques, results from the cleavage of its precursor, the amyloid precursor protein, while the neurofibrillary tangles are made up of hyperphosphorylated tau protein (Braak and Braak, 1995; Hardy and Selkoe, 2002; Goedert and Spillantini, 2006). Although these pathologies have a similar overall topography across the brain, being found in largely the same regions, they tend to have different patterns of development. The β-amyloid plaques appear first in the cortex and then later across subcortical regions, while hyperphosphorylated tau is first observed in the subcortex (e.g., locus coeruleus) and then later across the cortex (Brettschneider et al., 2015).

Debate concerning the cause of this dementia is robust. In the rare early-onset forms (<65 years), there are strong genetic links, with mutations of amyloid precursor protein or presenilins giving rise to an autosomal dominant inheritance pattern of the disease. The majority of the transgenic animal models of the disease are, in fact, based on mutations of these proteins (e.g., Garcia-Alloza et al., 2006; van Eersel et al., 2010). In the more common late-onset forms (>65 years), genetic associations are not as strong, with the underlying causes and mechanisms being unclear (Coppedè and Migliore, 2015; Goedert, 2015; Herrup, 2015). A number of different hypotheses have been championed, the most popular of which is the amyloid cascade hypothesis, which proposes that the accumulation of β-amyloid in the brain—whether by genetic mutation or other unknown factors—is the primary driver of pathogenesis, namely the formation of tangles and subsequent neuronal death (Hardy and Selkoe, 2002). In more recent times, Alzheimer's pathogenesis has been proposed to be generated by protein assemblies adopting alternative conformations and becoming self-propagating, like prions (Recasens et al., 2014; Brettschneider et al., 2015; Goedert, 2015). An alternative hypothesis suggests that the proteinopathies occur downstream from the prime cause, which is microvascular hemorrhage (Cullen et al., 2005, 2006; Stone, 2008). In this latter view, Alzheimer's disease is a vasculopathy, a form of vascular dementia (De la Torre, 2004). In essence, this hypothesis proposes that the breakdown of cerebral capillaries as a consequence of aging results in microhemorrhages that in turn lead to the formation of plaques, tangles, and subsequent neuronal death (Cullen et al., 2005, 2006; Stone, 2008). We have argued recently that the dementia is best understood as a pulse-induced vascular dementia affecting primarily small cerebral vessels and that the link to age arises from the age-related hardening of the aorta, which intensifies the destructiveness of the pulse; that the pathology and symptoms of the disease are all downstream outcomes of pulse-induced damage to cerebral vessels (Stone et al., 2015). Finally, there is the hypothesis that mitochondrial dysfunction is a major contributor to the neuronal death (Swerdlow and Khan, 2004; Chaturvedi and Beal, 2008; Gonzalez-Lima et al., 2014; Coppedè and Migliore, 2015). As the organelles responsible for fuelling cell function, if mitochondria become damaged or dysfunctional, their efficacy and ATP (adenosine triphosphate) yield would be reduced. This process would lead to an increase in toxic reactive oxygen species, generating oxidative stress and subsequent neuronal death, as observed in Alzheimer's disease (Swerdlow and Khan, 2004; Chaturvedi and Beal, 2008; Gonzalez-Lima et al., 2014; Coppedè and Migliore, 2015). It should be noted that each of these putative pathogenic processes need not be mutually exclusive, and that all probably play some role in the disease process (Stone et al., 2015).

The current treatment options for patients with Alzheimer's disease are limited. These include acetylcholinesterase inhibitors (AChEIs) and N-methyl-d-aspartate receptor (NMDA) antagonists. AChEIs work to slow the rate of cognitive decline by inhibiting the degradation of acetylcholine, the major neurotransmitter associated with attention and memory, while NMDA antagonists work to prevent neurotoxicity in the brain, in particular in regions that are important for memory formation and learning. Unfortunately, these drugs are not efficacious in most patients, may have some toxic side effects and at best provide only minor palliative symptomatic relief (Nelson and Tabet, 2015).

### Parkinson's disease

The clinical syndrome and neuropathology of Parkinson's disease are very different to Alzheimer's disease. Parkinson's patients have predominately motor signs, including resting tremor, lead-pipe rigidity, akinesia, and/or bradykinesia (Bergman and Deuschl, 2002; Jankovic and Poewe, 2012). There may also be some cognitive impairment but this generally develops very late in the disease process (Cosgrove et al., 2015). Unlike Alzheimer's patients, there are no plaques or tangles and the zones of neurodegeneration are more limited, at least initially. In Parkinson's patients, there is a progressive death of many neurons in the brainstem, in particular the dopaminergic cells in the substantia nigra pars compacta (SNc) of the midbrain (Figure 1; Rinne, 1993; Blandini et al., 2000; Bergman and Deuschl, 2002). The loss of these cells leads subsequently to a reduction in the levels of dopamine in the striatum that, in turn, manifests as the distinct signs of the disease (Blandini et al., 2000; Bergman and Deuschl, 2002). In addition to this primary loss of brainstem dopaminergic cells, there are also localized regions of pathology in the olfactory bulb, dorsal motor nucleus of the vagus nerve and locus coeruleus (Figure 1) and in much later stages of the disease, across the cortex (Del Tredici and Braak, 2013; Brettschneider et al., 2015).

As with Alzheimer's disease, the factors that cause Parkinson's disease and mechanisms of neuronal death are not clear. In the rarer, early-onset forms of Parkinson's disease (10–15%), strong genetic links have been established, with several gene mutations having been identified (e.g., parkin, PINK1; Corti and Brice, 2013). There are many transgenic animal models of the disease, the most relevant involving mutations of the presynaptic protein, α-synuclein (Blesa et al., 2012; Bezard et al., 2013). In the more common late-onset of forms of the disease, the genetic links are much weaker and the causes remain unknown. As with Alzheimer's disease, several hypotheses have been championed. First, the abnormal accumulation α-synuclein in cells (synucleinopathy)—whether by genetic mutation or other unknown factors—has been suggested to be the primary factor driving the neuronal death (Gitler et al., 2009). The abnormal accumulation of this protein in cells (i.e., Lewy bodies) is thought to have prion-like propagation (Brettschneider et al., 2015; Goedert, 2015). Second, there is evidence that Parkinson's disease arises after exposure to a neurotoxin, for example paraquat, rotenone, 6OHDA (6 hydroxydopamine) or MPTP (methyl-4-phenyl-1,2,3,6-tetrahydropyridine). Indeed, many of the animal models of the disease are based on exposure to these toxins (Blesa et al., 2012). Third, there are reports proposing a role for vascular dysfunction in Parkinson's pathogenesis. In particular, it has been suggested that the process of neuronal death begins after endothelial cell damage and impairment of blood-brain barrier function (Farkas et al., 2000; Kortekaas et al., 2005; Carvey et al., 2009; Grammas et al., 2011). Further, the toxins that induce parkinsonism in animal models, namely 6OHDA and MPTP, have been shown to generate substantial disruption of the blood-brain barrier (Carvey et al., 2009). Finally, mitochondrial dysfunction—caused by either toxic insult, genetic mutation, vascular damage, or unknown factors—is considered central in the pathogenesis of Parkinson's disease (Fukae et al., 2007; Exner et al., 2012). This dysfunction leads to a reduction of key cellular functions and subsequent neuronal death (see above). Many of these proposed mechanisms of neuronal death—from mitochondrial dysfunction to vascular compromise and from abnormal protein assemblies to prion-like propagation—are similar to those described above for Alzheimer's disease and are likely to all contribute to the pathological process, not being mutually exclusive.

For Parkinson's patients, there are more treatment options available than for Alzheimer's patients. Most Parkinson's patients are treated initially with dopamine replacement drug therapy, which aims to replace the dopamine lost from the system. This therapy is highly efficacious at reducing motor signs initially, but with prolonged use, its efficacy tapers and side-effects develop (e.g., dyskinesias; Bergman and Deuschl, 2002; Jankovic and Poewe, 2012). At these stages, patients are usually recommended for surgery with high frequency deep brain stimulation, most commonly targeting the subthalamic nucleus (Benabid et al., 2009). This surgery aims to correct the abnormal function of the basal ganglia circuitry caused by the loss of dopamine and, as with the drug therapy, is very effective in treating the signs of the disease. However, for both dopamine drug therapy and surgery, there is little, if any, evidence for neuroprotection in Parkinson's patients (Olanow et al., 2008; Jankovic and Poewe, 2012; Bezard et al., 2013; Schapira et al., 2014).

In summary, the neuropathology and patterns of neurodegeneration across the brain in Alzheimer's and Parkinson's disease are very different, hence resulting in very different signs and symptoms. However, there are similarities in the proposed mechanisms of neuronal death in each disease. The current treatments for patients of both diseases offer at best symptomatic relief (particularly in Parkinson's disease) but do not provide neuroprotection or are not disease-modifying, at least in humans.

## From the bench to the clinic: the evidence for neuroprotection by near infrared light (NIr) treatment in Alzheimer's and Parkinson's disease

Low-level laser or LED (light emitting diode) therapy using red to infrared light (λ = 600–1070 nm), conflated here to the term “near infrared light” (NIr), is an emerging, putative neuroprotective treatment that is showing promise in several pre-clinical models of disease. For example, NIr has been reported beneficial in animal models of retinal disease (Eells et al., 2004; Natoli et al., 2010, 2013; Albarracin et al., 2013; Begum et al., 2013; Gkotsi et al., 2014), traumatic brain (Ando et al., 2011; Oron et al., 2012; Quirk et al., 2012a; Xuan et al., 2013, 2014, 2015) and optic nerve (Fitzgerald et al., 2010) injury, experimentally-induced stroke (Lapchak et al., 2004; DeTaboada et al., 2006; Oron et al., 2006), familial amyotrophic lateral sclerosis (Moges et al., 2009), multiple sclerosis (Muili et al., 2012), Parkinson's disease (Liang et al., 2008; Whelan et al., 2008; Ying et al., 2008; Shaw et al., 2010; Peoples et al., 2012; Moro et al., 2013, 2014; Purushothuman et al., 2013; Vos et al., 2013; Johnstone et al., 2014a,b; Darlot et al., 2015; El Massri et al., 2015; Reinhart et al., 2015a,b) and Alzheimer's disease (Michalikova et al., 2008; DeTaboada et al., 2011; Grillo et al., 2013; Purushothuman et al., 2014, 2015). In humans, NIr therapy has been reported to improve executive, cognitive, and emotional functions (Barrett and Gonzalez-Lima, 2013; Blanco et al., 2015), together with performance in a range of clinical tests after ischaemic stroke (Lampl et al., 2007; Lapchak, 2010), brain trauma (Naeser et al., 2011, 2014), depression (Schiffer et al., 2009) and in age-related macular degeneration (Merry et al., 2012). The fact that NIr therapy has been reported to be effective in so many different models of disease and in a range of neural systems suggests that it is not a targeted therapy, but instead, acts to mitigate ubiquitous processes relating to cell damage and death. Recent work indicates that NIr is effective in reducing neuronal death induced by apoptosis, but not necrosis (Quirk et al., 2012a). The pathway to apoptosis is likely to involve a critical decline in cellular energy production (Galluzzi et al., 2012), that NIr may help to restore (Hamblin and Demidova, 2006; Liang et al., 2008; Ying et al., 2008; Desmet et al., 2009; Rojas and Gonzalez-Lima, 2011; Chung et al., 2012; Begum et al., 2013; Gkotsi et al., 2014). This mechanism is presumably common to all the above mentioned conditions and is perhaps why NIr therapy has such broad potential applications. In the context of Alzheimer's and Parkinson's disease, although they have distinct initiating causes, both diseases converge on common pathways of inflammation and oxidative stress, mitochondrial dysfunction and neuronal death, indicating that NIr may be beneficial to both through the same protective mechanisms.

### NIr for Alzheimer's disease

The majority of the studies reporting beneficial effects of NIr treatment in Alzheimer's disease or dementia have been in transgenic animal models, in particular those displaying β-amyloid (APP/PS1: DeTaboada et al., 2011; Purushothuman et al., 2014, 2015; TASTPM; Grillo et al., 2013; CD1: Michalikova et al., 2008), or tau (K369I: Purushothuman et al., 2014, 2015) pathologies (Table 1). In general, with either acute (weeks; Michalikova et al., 2008) or more chronic (months; DeTaboada et al., 2011; Grillo et al., 2013; Purushothuman et al., 2014, 2015) NIr treatment, these studies have reported reductions in β-amyloid plaques, neurofibrillary tangles of hyperphosphorylated tau protein, inflammation and oxidative stress, together with increased ATP levels and improved overall mitochondrial function. In addition, NIr reduced the characteristic cognitive deficits associated with the CD1 (Michalikova et al., 2008) and APP/PS1 (DeTaboada et al., 2011) transgenic mouse models. One *in vitro* study reported that, after internalization of β-amyloid into human neuroblastoma cells, NIr treatment increased ATP levels and overall cell number, while reducing β-amyloid aggregates (Sommer et al., 2012).

**Table 1:** **Studies reporting on NIr treatment in Alzheimer's disease**.

**Findings with NIr application**	**Study**	**Model**	**Species**
↑ Cell survival	Sommer et al., 2012	*In vitro* (neuroblastoma cells internalized with β-amyloid)	Human cells
↑ ATP content			
↓β-amyloid aggregates			
↓β-amyloid plaques	Purushothuman et al., 2014, 2015	APP/PS1, K3691 transgenics (chronic)	Mouse
↓ Oxidative stress			
↓ hyperphosphorylated tau			
↓β-amyloid plaques	DeTaboada et al., 2011	APP transgenic (chronic)	Mouse
↓ Inflammation			
↑ ATP content			
↑ Mitochondrial function			
↓β-amyloid plaques	Grillo et al., 2013	TASTPM transgenic (chronic)	Mouse
↓ Oxidative stress			
↓ Hyperphosphorylated tau			
↑ Heat shock proteins			
↑ Cognitive behavioral deficits	Michalikova et al., 2008	CD1 transgenic (acute)	Mouse
	DeTaboada et al., 2011	APP transgenic (chronic)	

To the best of our knowledge, there have been no major publications—at least in peer-reviewed journals—on the efficacy of NIr in Alzheimer's patients. There are some web pages referring to either an Alzheimer extracranial “helmet,” housing many LEDs of wavelengths ranging from 660 to 1070 nm (e.g., http://www.emersonww.com/InfraredHelmet.htm; http://www.science20.com/news_releases/can_this_infra_red_helmet_cure_alzheimers_in_10_minutes_a_day; http://www.instructables.com/id/LED-helmet-for-dementia-alzheimers-parkinsons), or an intranasal device delivering NIr to the brain (http://www.mediclights.com/wp-content/uploads/2013/11/Alzheimer-with-intranasal-light-08-22-13-1.pdf). However, there are no reports, either published, or in progress, of clinical trials on Alzheimer's patients. Two clinical studies by Naeser et al. (2011, 2014) have reported improvements in executive function, learning and memory after NIr treatment—delivered via an extracranial helmet-like device using two LEDs—in a small number of patients suffering chronic traumatic brain injury. Further, there are two human studies in healthy individuals reporting that NIr therapy improves attention and short-term memory (Barrett and Gonzalez-Lima, 2013) and executive functions (Blanco et al., 2015). Although these studies are promising in the sense that NIr therapy resulted in cognitive improvements, the subjects were not Alzheimer's patients.

### NIr for Parkinson's disease

Mainly due to the existence of effective toxin-based *in vitro* and *in vivo* models, there have been considerably more reports on the beneficial effects of NIr for Parkinson's disease (Table 2). The first studies to report neuroprotection by NIr after parkinsonian insult demonstrated that NIr treatment reduced cell death, increased ATP content and decreased levels of oxidative stress in rat striatal and cortical cells exposed to the parkinsonian toxins rotenone and MPP^+^ (1-methyl-4-phenylpyridium) *in vitro* (Liang et al., 2008; Ying et al., 2008). In cultures of human neuroblastoma cells engineered to overexpress α-synuclein, NIr increased mitochondrial function and reduced oxidative stress after MPP^+^ (1-methyl-4-phenylpyridinium) exposure (Trimmer et al., 2009; Quirk et al., 2012b). Further, in hybrid cells bearing mitochondrial DNA from Parkinson's patients, mitochondrial movement along axons improved substantially after NIr treatment, with movement restored to near control levels (Trimmer et al., 2009).

**Table 2:** **Studies reporting on NIr treatment in Parkinson's disease**.

**Findings with NIr application**	**Study**	**Model**	**Species**
↑ Cell survival (striatal and cortical cells)	Liang et al., 2008; Ying et al., 2008	*In vitro* (rotenone, MPTP)	Rat cells
↑ ATP content			
↓ Oxidative stress			
↑ Mitochondrial function	Quirk et al., 2012b	*In vitro* (neuroblastoma cells overexpressing <-synuclein)	Human cells
↓ Oxidative stress			
↑ mitochondrial movement	Trimmer et al., 2009	*In vitro* (hybrid cells with mitochondrial DNA from Parkinson's disease patients)	Human cells
↑ Cell survival (TH^+^ cells)	Shaw et al., 2010	MPTP (acute)	Mouse
↑ Cell survival (TH^+^ cells)	Peoples et al., 2012	MPTP (chronic)	
↑ Cell survival (TH^+^ cells)	Purushothuman et al., 2013	K369I transgenic (chronic)	
↑ Cell survival (TH^+^ cells)	Moro et al., 2013, 2014; Johnstone et al., 2014b	MPTP (acute)	
↑ Cell survival (TH^+^ cells)	El Massri et al., 2015	MPTP (acute, sub-chronic)	
↑ Cell survival (TH^+^ cells)	Reinhart et al., 2015b	MPTP (acute)	
↑ Cell survival (TH^+^ cells)	Reinhart et al., 2015a	6OHDA hemi-parkinsonian	Rat
↑ Cell survival (TH^+^ and Nissl-stained cells)	Darlot et al., 2015	MPTP (sub-acute)	Monkey
↓ Oxidative stress	Purushothuman et al., 2013	K369I transgenic (chronic)	Mouse
↓ Hyperphosphorylated tau			
↑ Flight	Vos et al., 2013	pink1 mutant	Flies
↑ Complex IV-dependent respiration			
↓ Mutant mitochondria defects			
↓ Abnormal basal ganglia activity (Fos immunoreactivity)	Shaw et al., 2012	MPTP (acute)	Mouse
↑ Locomotive behavior	Whelan et al., 2008	MPTP (acute)	Mouse
	Desmet et al., 2009	MPTP (acute)	
	Quirk et al., 2012b	A53T(<-synuclein transgenic)	
	Moro et al., 2013; Reinhart et al., 2015b	MPTP (acute)	
↓ Apomorphine-induced rotations	Reinhart et al., 2015a	6OHDA hemi-parkinsonian	Rat
↑ Locomotive behavior, clinical signs	Darlot et al., 2015	MPTP (sub-acute)	Monkey
↓ Clinical signs	Zhao et al., 2003; Maloney et al., 2010; Burchman, 2011	Parkinson's patients	Human
	Quietmind Foundation trial (http://www.youtube.com/watch?v=9X-hjgay7pg)		

There have also been many *in vivo* studies of NIr-induced neuroprotection in various animal models of Parkinson's disease (Table 2). In MPTP-treated mice (Shaw et al., 2010; Peoples et al., 2012; Moro et al., 2013, 2014; Johnstone et al., 2014b; El Massri et al., 2015; Reinhart et al., 2015b) and 6OHDA-lesioned rats (Reinhart et al., 2015a), NIr treatment saved many dopaminergic cells from death. Further, results were similar whether the therapy was applied before, at the same time or well after the insult, indicating that NIr both conditions healthy neurons to resist a subsequent insult and rescues damaged neurons following an insult (Peoples et al., 2012). The rescue of neurons is particularly relevant to the clinical reality of the parkinsonian condition, in which individuals have, at presentation, already suffered significant degeneration, so that treatment follows neuronal loss. In the K369I transgenic mouse model of frontotemporal dementia, which also shows parkinsonian signs and a chronic and progressive degeneration of dopaminergic cells in the SNc, NIr treatment decreased oxidative stress and hyperphosphorylated tau and increased dopaminergic cell survival in the SNc (Purushothuman et al., 2013). Recently, NIr therapy has been used in a non-human primate MPTP model of Parkinson's disease with very promising results. All of the NIr-treated MPTP monkeys had a greater number of surviving dopaminergic nigral cells and striatal terminations compared to those that were not treated (Darlot et al., 2015).

Together with preserving dopaminergic cell survival, NIr has been shown to correct abnormal neuronal activity generated by the parkinsonian condition (Shaw et al., 2012). Using Fos immunohistochemistry (a well-established measure of cell activity), the overactivity of neuronal firing in the subthalamic region, characteristic of parkinsonian cases, was reduced substantially after NIr therapy. This reduction did not quite reach control levels, indicating that the restoration was partial, and was attributed to the functional repair of damaged dopaminergic cells in the SNc, allowing these cells to resume producing and releasing dopamine at their terminals in the striatum (Shaw et al., 2012). This functional restoration may well-underlie the improved motor behavior observed after NIr treatment (see below).

A number of previous studies have reported clear improvements in motor behavior in animal models of Parkinson's disease following NIr treatment. In MPTP-treated mice, NIr therapy improved various parameters of locomotion, for example mobility, and velocity (Whelan et al., 2008; Moro et al., 2013; Reinhart et al., 2015b). NIr treatment also delayed disease progression and reduced the severity of the disease phenotype in transgenic mice expressing the A53T human α-synuclein mutation (Quirk et al., 2012b). Further, NIr treatment reduced apomorphine-induced rotational behavior in a 6OHDA-lesioned hemiparkinsonian rat model (Reinhart et al., 2015a). There is also evidence that NIr treatment rescues flight and mutant mitochondria defects, together with promoting complex IV-dependent respiration, in pink1 mutant flies (Vos et al., 2013). Perhaps the strongest evidence for improved behavioral outcomes after NIr treatment has been in the MPTP-treated monkey model of the disease. The NIr-treated MPTP monkeys all had reduced clinical signs compared to untreated MPTP monkeys; these reductions in clinical signs were still evident well after the period of NIr treatment, in fact up to 3 weeks after in many of the cases. This indicates that the therapeutic effects of NIr are long-lasting and not confined to periods when NIr is being applied (Darlot et al., 2015).

As with Alzheimer's disease, there have been few reports to date on the efficacy of NIr treatment in Parkinson's disease patients (Table 2). From the Quietmind Foundation trial, there is a linked YouTube video (http://www.youtube.com/watch?v=9X-hjgay7pg) of a Parkinson's patient displaying improved movement and reduced tremor after extracranial application of NIr, but few details are provided. There is a recent non-controlled and non-randomized clinical report indicating improved speech, cognition, freezing episodes and gait after extracranial NIr therapy in parkinsonian patients (Maloney et al., 2010); there are also some clinical reports suggesting improvements in parkinsonian signs in the majority of patients after NIr application through an intranasal device (Zhao et al., 2003). Finally, there is a serendipitous finding in one Parkinson's patient that was treated with NIr for a dental problem. This patient was reported to display a reduction in his parkinsonian signs following NIr treatment to the posterior regions of the cranium/upper neck (Burchman, 2011).

In summary, a number of experimental studies have demonstrated that NIr therapy improves motor behavior and provides neuroprotection in various rodent models of both Alzheimer's and Parkinson's disease; for Parkinson's disease, these benefits have been reported in a non-human primate model as well. However, the evidence for therapeutic benefit at the clinical level is far sparser, prompting the need for systematic, large-scale clinical trials of NIr therapy in Alzheimer's and Parkinson's patients.

## How does NIr work to neuroprotect?

**Figure 2:**
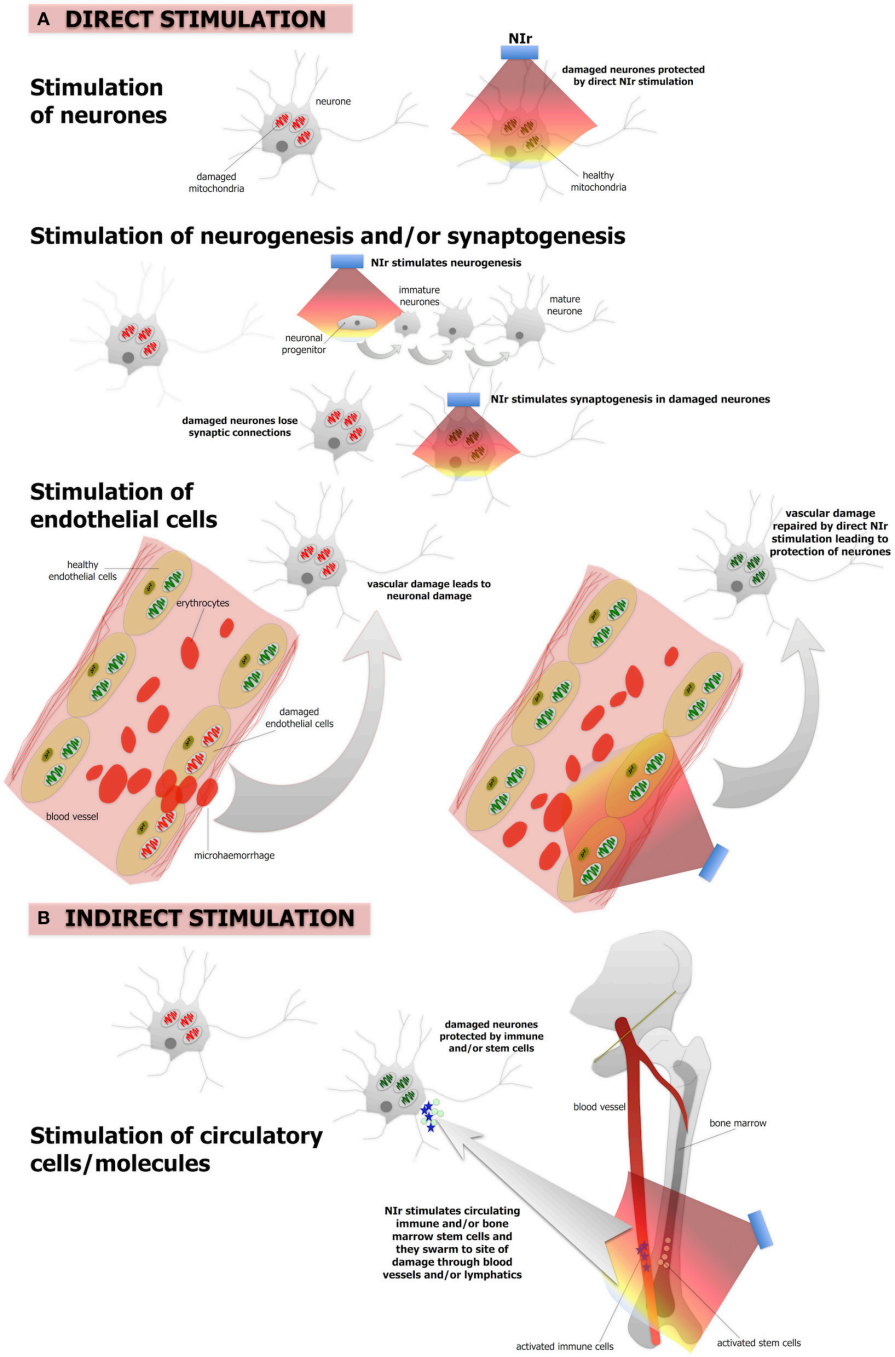
**The putative NIr protective mechanisms in the brain. (A)** Direct NIr stimulation of the mitochondria of the damaged neurons or endothelial cells. This stimulation would repair the damage leading to neuronal protection. NIr may also stimulate neurogenesis in the hippocampus and/or synaptogenesis in the damaged neurons **(B)** indirect (remote) stimulation via circulating immune cells and/or bone marrow stem cells leading to neuronal protection. The latter is similar to the so-called “abscopal” effect in the treatment of cancer metastasis. We suggest that the primary mechanism is the direct effect, of neurons and/or of endothelial cells, while the systemic indirect effect forms a secondary supportive mechanism.

The mechanisms that underpin NIr-induced neuroprotection are not entirely clear, although they appear to operate in at least two different biological levels. First, NIr acts at a cellular level, activating intracellular cascades that ultimately contribute to the survival of the target, and possibly neighboring, cells and/or stimulating neurogenesis. Second, NIr appears capable of triggering systemic protective mechanisms; this presumably involves as yet unidentified circulating cellular or humoral factors that can transduce protective effects to the brain (Figure 2).

### Direct stimulation of cells

There is a large body of work reporting that a number of molecular and cellular systems are influenced by NIr. At a cellular level, NIr displays a biphasic dose-response curve, suggesting that NIr is a low-level stressor of cells and that the activation of endogenous cellular stress response systems is likely to be central to its efficacy (Hamblin and Demidova, 2006; Desmet et al., 2009; Rojas and Gonzalez-Lima, 2011; Chung et al., 2012). The main direct target of NIr appears to be cytochrome c oxidase, a key enzyme of the mitochondrial respiratory chain (Figure 2A). This enzyme is a photoacceptor of light in the NIr range; NIr exposure produces a redox change in cytochrome c oxidase which causes a transient change in mitochondrial membrane potential, leading to increase ATP production and a burst in low levels of reactive oxygen species (Hamblin and Demidova, 2006; Desmet et al., 2009; Rojas and Gonzalez-Lima, 2011; Chung et al., 2012). This, in turns, triggers a cascade of secondary downstream signaling pathways that collectively stimulate endogenous cell protection and repair mechanisms (Hamblin and Demidova, 2006; Desmet et al., 2009; Chung et al., 2012; Rojas and Gonzalez-Lima, 2011). This modulation of multiple molecular systems appears capable of both conditioning neurons to resist future damage and accelerating repair of neurons damaged by a previous or continuing insult (e.g., Liang et al., 2008; Ying et al., 2008).

In addition to protecting and repairing damaged or dysfunctional neurons, there is emerging evidence from mouse models of traumatic brain injury that NIr also stimulates neurogenesis and synaptogenesis (Figure 2A). In a series of studies using a mouse model of traumatic brain injury, Xuan and colleagues found that a NIr treatment regime that improved neurological performance (Xuan et al., 2013), also increased markers of neuroprogenitor proliferation in the hippocampal region (i.e., dentate gyrus) and subventricular zone (Xuan et al., 2014), brain regions known to harbor neural stems cells. Other early responses in these regions included up-regulation of brain-derived neurotrophic factor, which was associated with subsequent up-regulation of synaptogenesis markers in the lesion site (Xuan et al., 2013). Similar observations of NIr-induced increases in neuroprogenitor cell proliferation in the subventricular zone have been made in a rat model of stroke (Oron et al., 2006).

It should be noted that these studies have focussed on the effect of NIr on neurons; similar NIr-induced cellular mechanisms may also be at play within brain capillary endothelial cells (Figure 2A). Mitochondrial dysfunction of these cells has been related to various vascular conditions, including atherosclerosis and hypertension (Tang et al., 2014). In the context of neurodegeneration, both Alzheimer's and Parkinson's disease have been implicated as vascular disorders, with suggestions that the neurodegenerative process begins with the breakdown of the integrity of small cerebral vessels and the blood-brain barrier (see above). This “breakdown” may begin with mitochondrial dysfunction (Grammas et al., 2011). Following, we propose that NIr-induced neuroprotection in Alzheimer's and Parkinson's disease might involve repair of the damaged mitochondria in local endothelial cells, leading subsequently to a restoration of the integrity of the endothelial network and blood-brain barrier in the region, resulting ultimately in improved neuronal survival (Figure 2A).

### Indirect stimulation of systemic factors

In addition to direct beneficial actions on damaged cells, there is increasing evidence that NIr treatment might also activate a more global, systemic response (Figure 2B). This evidence arises from the observation that local application of NIr to a particular body part can induce beneficial effects in distant body tissues (Braverman et al., 1989; Stone et al., 2013; Johnstone et al., 2014a,b, 2015). For example, neuroprotection of the mouse brain against MPTP insult has been demonstrated following the “remote” application of NIr to the dorsum of the animal, with no direct application to the head (Stone et al., 2013; Johnstone et al., 2014a,b, 2015). While the mechanism remains unknown, it presumably involves the stimulation of one or more circulating molecules or cell types. One possibility is the stimulation of immune cells, for example mast cells and macrophages, that could help neuroprotect cells in the brain (Byrnes et al., 2005; Chung et al., 2012; Muili et al., 2012). There may also be effects on inflammatory mediators, as NIr is associated with down-regulation of pro-inflammatory cytokines and up-regulation of anti-inflammatory cytokines (Muili et al., 2012). In addition, bone marrow-derived stem cells may also be involved; a series of studies has demonstrated that NIr exposure increases proliferation of c-kit-positive cells in the bone marrow and that, following myocardial infarction in rats, these cells are mobilized and recruited specifically to the site of damage where they are associated with a reduction in myocardial infarct size and ventricular dilatation (Tuby et al., 2011). These cells, together with immune cells, may release trophic factors (e.g., nerve growth factor, brain-derived neurotrophic factor) that improve the function of dying cells and help their survival (Hou et al., 2008).

Another possibility is for a signaling system between mitochondria in different body tissues. Mitochondria in distress in one body tissue have been suggested to produce an unidentified extracellular signal (mitokine) that is then transmitted to cells in remote body tissues and as a consequence induces a mitochondrial stress response (Durieux et al., 2011; Taylor et al., 2014). In relation to NIr and Alzheimer's and Parkinson's disease, NIr applied to remote tissue may prompt a signal system between mitochondria of peripheral tissues and brain, inducing repair mechanisms in the damaged cells in the brain (Johnstone et al., 2014a,b, 2015). Taken all together, the systemic mechanisms underlying remote NIr-induced neuroprotection may share similarities with other remote tissue protection phenomena—these include remote ischaemic conditioning, where induction of brief ischaemic episodes in one organ provides protection of other distant organs (Hausenloy and Yellon, 2008; Yetgin et al., 2012), and the so-called “abscopal” effect sometimes observed in radiation treatment of metastatic cancer, where treatment targeted at a tumor leads to not only a shrinking of the local tumor but also a shrinking of tumors far from the treated area (Postow et al., 2012).

More research is required to understand the interplay between direct cellular and indirect systemic mechanisms of NIr-induced protection. Both appear capable of acting independently—the findings of numerous *in vitro* cell culture studies reporting that NIr is neuroprotective, indicate clearly that the indirect systemic effect is not necessary for NIr-induced neuroprotection and repair of damaged neurons (Hamblin and Demidova, 2006; Desmet et al., 2009; Rojas and Gonzalez-Lima, 2011; Chung et al., 2012), while accumulating evidence from mouse models suggest remote NIr application provides neuroprotection in the absence of direct NIr stimulation (Johnstone et al., 2014b, 2015; Farfara et al., 2015). The phenomenon of indirect NIr-induced neuroprotection is likely to involve the same mechanisms, at a cellular level, as those that provide neuroprotection to damaged cells with direct NIr stimulation (i.e., stimulation of mitochondrial function; Figure 2A). Although the concept of indirect, remote NIr therapy holds promise for future applications, it is not yet as fully understood and developed as direct NIr therapy, thus our subsequent discussion will focus primarily on direct NIr stimulation. Further, some early results in an animal model of Parkinson's disease suggest that, although remote NIr provides neuroprotection, this protection was not as robust as when NIr was applied directly to the head (Stone et al., 2013; Johnstone et al., 2014b; presumably stimulating local neurons and/or endothelial cells). In other words, neuroprotection was achieved with both local and remote NIr treatment, but the local treatment was the more effective. As a working hypothesis, we suggest that direct stimulation of the mitochondria and reparative mechanisms, either in the neurons themselves or in the local endothelial cells (and/or stimulation of neurogenesis), forms the primary mechanism of NIr-induced neuroprotection. A more systemic (indirect) stimulation of immune and/or stem cells may form a secondary and complementary mechanism. We suggest that stimulation of both direct and indirect mechanisms would generate maximum NIr-induced neuroprotection.

## Is NIr therapy safe?

To date, there are no reports of major safety issues nor side-effects after NIr treatment. The commercial LED panels for NIr therapy have already received non-significant risk status by the Food and Drug Administration and previous studies have indicated no adverse impact on brain tissue structure and function after NIr treatment (power range from ~1 to 700 mW/cm^2^; Desmet et al., 2006; Hamblin and Demidova, 2006; Ilic et al., 2006; Zivin et al., 2009; McCarthy et al., 2010; Naeser et al., 2011, 2014; Rojas and Gonzalez-Lima, 2011; Chung et al., 2012; Tata and Waynant, 2012; Quirk et al., 2012a,b; Moro et al., 2014). There is one sole account of some neuronal damage and negative behavioral outcomes in mice, but this was evident after an exceptionally high power intensity (750 mW/cm^2^; Ilic et al., 2006), approximately one hundred times higher than the dose required to elicit a therapeutic response (e.g., < 10 mW/cm^2^). Hence, when taken together, these data indicate that when NIr was applied at therapeutic doses (and even well above these doses), its impact on body tissue was overwhelmingly positive, and had a very large safety margin of application (Desmet et al., 2006; Hamblin and Demidova, 2006; Ilic et al., 2006; Zivin et al., 2009; McCarthy et al., 2010; Naeser et al., 2011, 2014; Rojas and Gonzalez-Lima, 2011; Chung et al., 2012; Tata and Waynant, 2012; Quirk et al., 2012a,b; Moro et al., 2014). Further, there appears to be no longer-term side effects associated with NIr application; in a long-term study in rats, no adverse effects were noted after daily treatment for 12 months (McCarthy et al., 2010).

## NIr therapy in Alzheimer's and Parkinson's disease patients: Can it work?

The key question that still remains is whether NIr therapy can be neuroprotective in humans. In order for maximum effect, the primary goal would be for sufficient NIr signal to reach the main zones of pathology, to elicit a protective, or reparative effect within damaged cells (and perhaps also neurogenesis); a secondary goal would be for the NIr signal to also trigger systemic neuroprotective factors, for example circulating cells or molecules (see above).

The issue of NIr reaching the zones of pathology is of most concern in humans. There are no such concerns when there are few or no tissue barriers, as in the culture dish (Eells et al., 2004; Wong-Riley et al., 2005; Liang et al., 2008; Ying et al., 2008), the retina (Natoli et al., 2010, 2013; Albarracin et al., 2013; Begum et al., 2013) or in the mouse brain (Shaw et al., 2010; Peoples et al., 2012; Moro et al., 2013; Purushothuman et al., 2013, 2014, 2015; Johnstone et al., 2014b; El Massri et al., 2015; Reinhart et al., 2015b). But can NIr be effective when there are many intervening body tissues, namely skin, thick cranium, and meninges, and brain parenchyma, as in humans?

Previous studies have estimated that NIr can be measured—through body tissues—at a distance of 20–30 mm from the transmission source (Lapchak et al., 2004; Byrnes et al., 2005; Zivin et al., 2009), albeit with a considerable dissipation of signal (DeTaboada et al., 2006; Zivin et al., 2009; Shaw et al., 2010; Abdo et al., 2013; Moro et al., 2014). For example, Moro et al. (2014) have noted that at a distance of 10 mm through brain parenchyma, the NIr signal is < 1% of that emitted from the source. They estimated a 65% reduction of signal across each millimeter of brain tissue.

**Figure 3:**
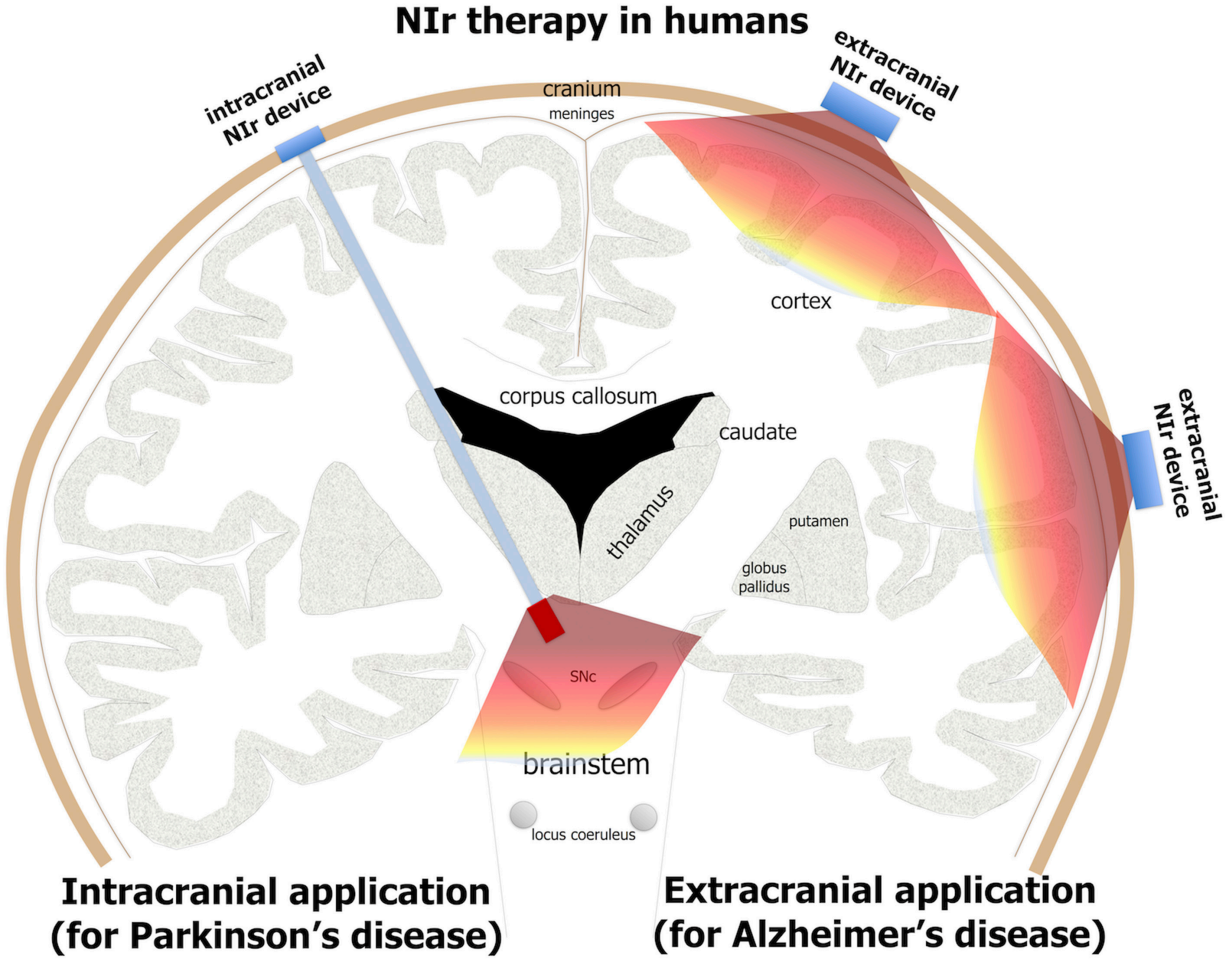
**Potential NIr applications in Alzheimer's and Parkinson's patients**. For effective neuroprotection, NIr could be applied extracranially in Alzheimer's disease (e.g., in the form of a helmet) and intracranially in Parkinson's disease (e.g., in the form of an optical fiber linked to a LED or laser source). NIr would be delivered very close to the diseased cells in the neocortex (for Alzheimer's) and brainstem SNc (for Parkinson's). In Parkinson's patients selected for deep brain stimulation, the NIr optical fiber could be implanted surgically at the same time, for neuroprotection of remaining dopaminergic cells (see text for details).

For Alzheimer's patients, the NIr signal—when applied from an extracranial source—should be able to reach the main zones of pathology located in the cortex, 8–10 mm below the cranium, and have therapeutic effects (Figure 3). Indeed, there have been several human studies reporting that NIr therapy is beneficial when the target region is in the cortex, for example in patients suffering trauma (Naeser et al., 2011, 2014), stroke (Lampl et al., 2007; Lapchak, 2010) or depression (Schiffer et al., 2009). Further, NIr therapy has been shown to improve higher-order cortical functions in healthy individuals, such as sustained attention and short-term memory (Barrett and Gonzalez-Lima, 2013), together with executive functions (Blanco et al., 2015). Hence, in Alzheimer's disease, NIr-induced neuroprotection appears feasible because the main zones of pathology are in superficial structures seemingly within reach from an extracranial source.

For Parkinson's patients, the distance from cranium to the main zone of pathology in the brainstem is much greater, being 80–100 mm (Johnstone et al., 2014a). Hence, it is unlikely that NIr signal from an extracranial source would reach the target cells (Figure 3); at these distances, the signal would be at best extremely weak and probably undetectable. This presents a clear limitation in the use of extracranially-applied NIr as a neuroprotective treatment in Parkinson's patients. For these reasons, we have developed a novel method of delivering effective NIr signal to deeper brainstem structures, using an intracranial optical fiber device. This device, when implanted within the brain parenchyma near the region of pathology, delivers NIr in effective doses for neuroprotection, for improved behavioral outcomes and with no toxicity to surrounding tissues in both rodents (Moro et al., 2014; Reinhart et al., 2015a) and non-human primates (Darlot et al., 2015).

We should note that in Parkinson's patients, although extracranially-delivered NIr may not reach the zones of pathology in the brainstem and hence, we argue, have limited neuroprotection, it may nevertheless provide some purely symptomatic effects. In Parkinson's disease, there is much abnormal activity in the cortex (Samuel et al., 1997; Sabatini et al., 2000; Haslinger et al., 2001), a structure that is within range of NIr signal when applied from an extracranial source (see above). NIr may help normalize this neural activity, leading to improvements in movement (Johnstone et al., 2014a). Here, the NIr therapy would impact on the abnormal neural circuitry that has resulted from the loss of dopaminergic cells, rather than on the diseased dopaminergic cells themselves. This form of NIr treatment would be purely symptomatic, rather than neuroprotective. We propose that such symptomatic treatment by NIr, namely clinical improvements without any underlying changes to the pathology, would be short-term; for long-lasting clinical improvements, we suggest that a reduction in the pathology through neuroprotection would be required. Hence, for neuroprotective and maximum therapeutic effects in Parkinson's disease, NIr would need to be applied via the intracranial optical fiber device (Figure 3).

In summary, there are clear indications that NIr can be an effective neuroprotective treatment for both neurodegenerative diseases, although the modes of delivery would be different; while extracranial NIr therapy would suffice for Alzheimer's disease, intracranial NIr therapy would be required for Parkinson's disease (Figure 3).

## What would be the advantages of using NIr therapy?

There would be several key advantages for the use of NIr therapy over current treatments for both Alzheimer's and Parkinson's disease. First and foremost, NIr has the potential to be neuroprotective. A growing body of pre-clinical evidence indicates that NIr therapy slows or stops disease pathology (Liang et al., 2008; Ying et al., 2008; Shaw et al., 2010; Peoples et al., 2012; Moro et al., 2013; Purushothuman et al., 2013, 2014, 2015; Johnstone et al., 2014b; El Massri et al., 2015; Reinhart et al., 2015a,b). This is something that the current mainstay of treatments for both diseases—drug therapy—does not do. Second, it is safe, with no reported side effects (see above). Third, treatment would be simple. For potential neuroprotection in Alzheimer's disease, patients would apply the NIr extracranially, perhaps in the form of a helmet or a hand held device, over the entire cranium; in Parkinson's disease, patients would require a minimally invasive surgical stereotactic procedure for the insertion of a NIr optical device within the brain; in some cases, this procedure might be undertaken at the same time as stereotactic surgery for deep brain stimulation (see below). This device would be linked to a battery source and pacemaker device (as with patients receiving deep brain stimulation; Benabid et al., 2009) applying the NIr to the brainstem when required. The procedural risks would be comparable to those of single electrode deep brain stimulation.

## Conclusions and implications of future therapy

Although in its infancy, with the bulk of results still at the pre-clinical “proof of concept” stage, NIr therapy has the potential to develop into a safe and effective neuroprotective treatment for patients with Alzheimer's and Parkinson's disease (and presumably other neurodegenerative diseases such multiple sclerosis and amyotrophic lateral sclerosis). If NIr was applied at early stages of the disease process, for example at first diagnosis, it could potentially slow further progression by protecting neurons from death. Consequently, over time, the greater neuronal survival would lessen the clinical signs and symptoms. Further, NIr therapy—because of its lack of side-effects and neuroprotective potential—is amenable to use in conjunction with other treatments. For example, patients may have NIr therapy with a reduced dosage of drugs as a first line treatment; the potential neuroprotective effect of NIr could prolong the efficacy of the drug therapy. Further, in Parkinson's patients selected for deep brain stimulation, they may also have an NIr optical fiber implanted surgically at the same time, thereby potentially offering neuroprotection of the remaining dopaminergic cells. There is much to do in further developing this treatment, but the therapeutic possibilities are many and the potential outcomes very exciting. We await the outcomes of major clinical trials using NIr therapy on these patients with much anticipation.

## Author contributions

DJ, CM, JS, AB, and JM are members of staff at their respective institutions. All authors contributed to the design and writing of the manuscript.

### Conflict of interest statement

The authors declare that the research was conducted in the absence of any commercial or financial relationships that could be construed as a potential conflict of interest.

## Abbreviations

AchEIs: acetylcholinesterase inhibitors
ATP: adenosine triphosphate
LED: light emitting diode
MPP^+^: 1-methyl-4-phenylpyridinium
MPTP: methyl-4-phenyl-1,2,3,6-tetrahydropyridine
NIr: near infrared light
NMDA: N-methyl-d-aspartate receptor
SNc: substantia nigra pars compacta
6OHDA: 6 hydroxydopamine.
